# Supplementary material for: Study on spatial distribution, regional differences and dynamic evolution of rural financial risk in China
Source: PLoS One. 2024 May 20;19(5):e0301977. doi: 10.1371/journal.pone.0301977 (PMC11104626; doi:10.1371/journal.pone.0301977)
Supplement: Attachment — Submitted filename: PONE-D-23-36724=RR.docx [file pone.0301977.s003.docx]

**Spatial Distribution, Regional Differences and Dynamic Evolution of Rural Financial Risk in China**

**Reviewer comments:**

The approach for evaluating financial risk in rural areas is not well defined in the article. To guarantee validity and repeatability, the authors should include a more thorough description of the variables and indicators they employed in their research.

This suggests that not all pertinent facets of rural financial risk may have been sufficiently covered by the statistical techniques employed in the investigation. It implies that a more comprehensive or in-depth approach to the investigation would be advantageous.

The recommendation to use more complex statistical tools, such spatial econometrics or time-series analysis, suggests that these approaches may provide a more comprehensive and nuanced understanding of financial risk in rural areas. While spatial econometrics takes into account the geographical interactions between variables in addition to their temporal dynamics, time-series analysis concentrates on analysing data points gathered over time to detect trends and patterns.

Using more advanced statistical methods aims to produce a more reliable evaluation of financial risk in rural areas. Robust assessments are more likely to be accurate, thorough, and dependable, which might result in better policy recommendations and decision-making.

The study's possible data constraints are not sufficiently addressed in the report. It is crucial to recognise and address any data constraints that can have an impact on the validity and generalizability of the findings given the complexity of rural financial risk.

The literature review is shallow and superficial. To demonstrate the importance of their study in the larger academic framework, the authors ought to present a more thorough analysis of the body of work already written on rural financial risk in China, taking into account current advancements and discussions in the area.

The analysis's results are unduly basic and fall short of capturing the nuances of China's rural financial risk. It is recommended that the authors offer more comprehensive and perceptive analyses of their results, including the wider socioeconomic and policy ramifications.

It might be challenging for readers to understand the points made because of the writing style, which is frequently ambiguous and dense. In order to ensure that the main arguments are expressed clearly and succinctly, the authors should rewrite the document.

The study's theoretical foundation is not well defined or stated. The conceptual framework and theoretical presumptions that underpin the authors' analysis should be spelt out in detail, providing a more solid theoretical basis for the research.
