# Supplementary material for: Study on spatial distribution, regional differences and dynamic evolution of rural financial risk in China
Source: PLoS One. 2024 May 20;19(5):e0301977. doi: 10.1371/journal.pone.0301977 (PMC11104626; doi:10.1371/journal.pone.0301977)
Supplement: Attachment — Submitted filename: Response to Reviewers.docx [file pone.0301977.s004.docx]

**Study on spatial distribution, regional differences and dynamic evolution of rural financial risk in China**

Manuscript ID: PONE-D-23-36724

--Response to Reviews--

March 5, 2024

**Note to Editors and Reviewers**

Thank you for giving us the opportunity to revise our article. The comments raised during this review highlighted quite a few areas in need of revision. We have taken every effort to address the comments, which has enabled us to significantly improve the contributions and delivery of our paper.

We have reproduced your comments (in blue font type) so that they can be considered in juxtaposition with our responses. We have also numbered the comments to better organize our responses. Please note that in our responses to the comments, the references to text locations (e.g., formulas, chapter contents and so on) refer to the *Revised Manuscript with Track Changes*.

It’s worth mentioning that the formats of literature citation, chart, figure, title, reference, etc. have been standardized in the revised manuscript according to the format of *PLOS ONE*. The serial number of the cited references in this article is marked with yellow, and the other modified and added parts are marked with red text.

**Responses to Journal Requirements**

1. Please ensure that your manuscript meets PLOS ONE's style requirements, including those for file naming. The PLOS ONE style templates can be found at 
<https://journals.plos.org/plosone/s/file?id=wjVg/PLOSOne_formatting_sample_main_body.pdf>

and 
<https://journals.plos.org/plosone/s/file?id=ba62/PLOSOne_formatting_sample_title_authors_affiliations.pdf>.

**Response:** Yes! Thank you so much! We have been changed the formats of references, formulas, figures’ and tables’ titles, etc. so that meeting PLOS ONE’s style requirements. Please see the *Revised Manuscript with Track Changes* for details.

2. Note from Emily Chenette, Editor in Chief of PLOS ONE, and Iain Hrynaszkiewicz, Director of Open Research Solutions at PLOS: Did you know that depositing data in a repository is associated with up to a 25% citation advantage (<https://doi.org/10.1371/journal.pone.0230416>)? If you’ve not already done so, consider depositing your raw data in a repository to ensure your work is read, appreciated and cited by the largest possible audience. You’ll also earn an Accessible Data icon on your published paper if you deposit your data in any participating repository (<https://plos.org/open-science/open-data/#accessible-data>).

**Response:** Yes, you’re right! Thank you very much! I’m glad to deposit my raw data in a repository. And we have been uploaded relevant datasets along with the submission of the revised manuscript for reference purposes.

3. We note that your Data Availability Statement is currently as follows: All relevant data are within the manuscript and its Supporting Information files. Please confirm at this time whether or not your submission contains all raw data required to replicate the results of your study. Authors must share the “minimal data set” for their submission. PLOS defines the minimal data set to consist of the data required to replicate all study findings reported in the article, as well as related metadata and methods (<https://journals.plos.org/plosone/s/data-availability#loc-minimal-data-set-definition>). For example, authors should submit the following data:

- The values behind the means, standard deviations and other measures reported;

- The values used to build graphs;

- The points extracted from images for analysis.

Authors do not need to submit their entire data set if only a portion of the data was used in the reported study. If your submission does not contain these data, please either upload them as Supporting Information files or deposit them to a stable, public repository and provide us with the relevant URLs, DOIs, or accession numbers. For a list of recommended repositories, please see <https://journals.plos.org/plosone/s/recommended-repositories>. If there are ethical or legal restrictions on sharing a de-identified data set, please explain them in detail (e.g., data contain potentially sensitive information, data are owned by a third-party organization, etc.) and who has imposed them (e.g., an ethics committee). Please also provide contact information for a data access committee, ethics committee, or other institutional body to which data requests may be sent. If data are owned by a third party, please indicate how others may request data access.

**Response**: Thank you for your valuable suggestions! Yes, we have been shared relevant datasets supporting the conclusions of this paper and also uploaded them to supporting information files, please refer to *S1 Data.* and *S2 Data.* in the Supporting information on page 26 of the *Revised Manuscript with Track Changes*.

4. PLOS requires an ORCID iD for the corresponding author in Editorial Manager on papers submitted after December 6th, 2016. Please ensure that you have an ORCID iD and that it is validated in Editorial Manager. To do this, go to ‘Update my Information’ (in the upper left-hand corner of the main menu), and click on the Fetch/Validate link next to the ORCID field. This will take you to the ORCID site and allow you to create a new iD or authenticate a pre-existing iD in Editorial Manager. Please see the following video for instructions on linking an ORCID iD to your Editorial Manager account: <https://www.youtube.com/watch?v=_xcclfuvtxQ>.

**Response:** Yes, Thank you very much! Dr. He (the corresponding author of the article) has been created a new ID and uploaded the information.

**Responses to the Reviewer #1**

The author(s) have studied “Study on Spatial Distribution, Regional Differences and Dynamic Evolution of Rural Financial Risk in China”. The contents of the research are novel and have potential to be published after some minor comments.

**Response:** Thank you very much! Thank you for your appreciation and valuable comments. After carefully addressing your comments, we have made this paper much stronger.

**Specific comments:**

1. What is Spatial Distribution?

**Response:** Thank you for your question. Spatial distribution refers to the arrangement of objects, phenomena, or attributes in space, describing their spatial correlation and clustering characteristics in geographical distribution. It also encompasses the spillover effect and proximity characteristics to some extent [1]. The study of the spatial distribution of rural financial risk is a prerequisite for examining the impact of spatial correlation among different areas in China, which holds significant implications for the rural economy and the prevention and control of systemic financial risk. Please refer to the description in the second paragraph of the manuscript’ *Introduction*.

2. How is this study helpful to the economy of china?

**Response:** Thank you very much! The study of spatial spillover effects in the local financial system is instrumental in stabilizing regional economic development. Accurately measuring the degree of correlation between local financial risks can enhance the comprehensive supervision capabilities of government authorities, thereby preventing and resolving systemic financial risks [2]. *As a major agricultural nation, the robust development of China's rural economy holds immense significance for the advancement of its overall economic growth, with rural finance serving as a catalyst for this progress. However, it is important to acknowledge that while rural finance contributes to economic expansion, it also entails certain risks*. In order to better promote the development of rural economy in China, it’s crucial to assess the correlation and impact of rural financial risk across different regions. Moreover, attention should be given to regional disparities and the evolution of rural financial risks from a spatial perspective, with an aim to strengthen the management of agricultural, rural, and farmer-related financial risks.” On the basis of the first paragraph of the *Introduction*, we have made some textual supplements (see the red text for details on page 3) to enhance the logic of the story.

3. What are basic gaps which are filled in this research study?

**Response:** Thank you for your insightful question. We adopt a spatial perspective to analyze the variations in China's rural financial risk levels across different regions, which represents a novel research approach. This study employs diverse methodologies to systematically examine the spatial distribution, regional heterogeneity, and dynamic evolution characteristics of rural financial risk in China. Specifically, we first utilize the objective weighting method of entropy weight to construct the rural financial risk index for 30 provinces in China. Then, the Moran index is employed to assess the spatial agglomeration patterns of rural financial risk across various regions and their changes in risk levels. Again, we employ the Dagum Gini coefficient and kernel density estimation three-dimensional diagram to examine the relative and absolute disparities of rural financial risk in China, facilitating a comprehensive visual analysis. All in all, the aforementioned studies represent a commendable endeavor in the realm of rural financial risk analysis, and the acquisition of valuable empirical findings holds immense significance for the management of rural financial risk in China, thereby fostering the advancement of rural economy.

4. How is this study compared to the previously published? Which issues are addressed in this which are not in the previously?

**Response:** The question you raised is quite insightful, and I sincerely appreciate it. Different from the previous literature that primarily focused on micro-level analysis of risk management in rural financial institutions [3-4], this paper examines the characteristics of overall risk changes in rural finance in China from a meso and macro perspective. It further conducts an extensive study on the spatial correlation and regional heterogeneity of rural financial risk across different regions in China. Additionally, unlike previous studies that relied on relatively simplistic econometric methods and subjective weighting approaches [5] to construct the financial risk index, this paper adopts an objective weighting method for constructing the rural financial risk index. Then, following the methods of Moran index testing, Dagum Gini coefficient decomposition, and kernel density estimation analysis, we systematically study the spatial distribution, regional heterogeneity, and dynamic evolution characteristics of rural financial risk in China. Please refer to the final paragraph of *Literature Review* section on page 7.

Therefore, there remains a significant scope for further exploration in this field of studying rural financial risk. This paper systematically analyzes, and solves the following questions, including the different spatial disparities of rural financial risk across different regions in China, as well as the crucial aspects pertaining to the dynamic evolution characteristics, which are systemically analyzed in this paper. Please refer to the Second paragraph of *Introduction* section on page 3.

5. The ranges of all parameters should be given? In caption mention all of the involved pare metric values for which graphs are plotted, it will help to the reader compare the parametric effects for considered values of the parameters.

**Response:** Thank you for your valuable and insightful suggestions! We have been checked all tables’ data, parameters’ values and corresponding graphs presented in the paper, and added the series parametric values of *x*_4_, *x*_9_ and *x*_13_ reported in Table 2 (as shown on pages 14-15 of Table 2 of *Empirical Analysis* in the manuscript) so that helping readers comparing the parametric values. Furthermore, to support the research contents, we have been provided detailed information on data support, please refer to the *Support information* on page 27 of the manuscript.

6. Conclusion should be precise.

**Response:** Thank you for your insightful suggestion, we have been corrected it as much as possible to ensure the precise conclusions. Please refer to the corrected descriptions with red text in the results analysis of *Empirical Research* and also the final summary *Conclusions* section of the *Revised Manuscript with Track Changes*.

7. What specific improvements should the authors consider regarding the methodology? What further controls should be considered?

**Response:** Thank you for your insightful questions! We start from the perspective of spatial distribution of rural financial risk, but with the limitations of subjective weighting method and the single measurement research methods, after the inspiration of Liu et al.’ [6] research, we consider the following studying methods. Therefore, we employed the objective weighting method to construct rural financial risk index model, conducted Moran index test to analyze the spatial agglomeration characteristics, utilized Dagum coefficient and kernel density estimation three-dimensional figure to examine relative difference and absolute difference of rural financial risk in China, respectively. This paper always carries out research based on the constructed rural financial risk, synthetically analyzes a series of tests. We aim to comprehensively investigate the spatial distribution, regional heterogeneity, and dynamic evolution patterns of China's rural financial risk level, so that it’s possible to provide recommendations for rural financial risk management through a series of tests and analyses.

8. At the end novelty of the work should be highlighted clearly.

**Response:** Thank you for your useful comments again! We have been corrected it and highlighted the novelty of this paper, please see the red text in the first paragraph of the *Conclusions* section on page 24 of the *Revised Manuscript with Track Changes*.

**References**

[1] Wang SY, Sun B, Qing F. The Correlation between Rural Financial Development and Rural Economic Growth based on Spatial Panel Durbin Model. *Chinese Journal of Agricultural Resources and Regional Planning*. 2016;37(9):196-204. (in Chinese)
[2] Shen L, Liu Y, Li WJ. China’s Regional Financial Risk Spatial Correlation Network and Regional Contagion Effect: 2009-2016. *Management Review*. 2019; 31(8): 35-48. doi:10.14120/j.cnki.cn11-5057/f.2019.08.004. (in Chinese)
[3] Du YN, Hu JC. Constructing investment and financing system of new rural residential with new rural financial institutions as the core based on Hebei province. *Guangdong Agricultural Sciences*. 2012; 39(4): 215-216+223. doi:10.16768/j.issn.1004-874x.2012.04.045. (in Chinese)
[4] Yu J, Cui H. Rural Financial Decision Support System Based on Database and Genetic Algorithm. *Wireless Communications & Mobile Computing*. 2022; 2022: 9662953. doi:10.1155/2022/9662953.
[5] Liu ZL. Study on application of AHP in agricultural system. *Guangdong Agricultural Sciences*. 2013; 40(13): 228-232. doi:10.16768/j.issn.1004-874x.2013.13.002. (in Chinese)
[6] Liu FG, Liao ZJ, Zhang M. Research on the Spatial Distribution and Evolution Characteristics of Regional Financial Risks in China. *Journal of Yunnan University of Finance and Economics*. 2022; 38(4): 66-84. doi:10.16537/j.cnki.jynufe.000780. (in Chinese)

**Responses to the Reviewer #2**

The study's theoretical foundation is not well defined or stated. The conceptual framework and theoretical presumptions that underpin the authors' analysis should be spelt out in detail, providing a more solid theoretical basis for the research.

**Response:** Thank you for your highly valuable comments. After carefully addressing your comments, this manuscript has become much stronger and more friendly to the readers.

**Specific comments:**

1. The approach for evaluating financial risk in rural areas is not well defined in the article. To guarantee validity and repeatability, the authors should include a more thorough description of the variables and indicators they employed in their research.

**Response:** Very good comments. Thank you so much! Actually, the approach of constructing rural financial risk index is descripted in the ‘*Evaluating rural financial risk index*’ section on page 7. Furthermore, we have been provided additional theoretical descriptions of the indicators used in this paper to enhance its logical coherence. Please see the revised parts indicated by red texts in ‘*Indicators selection and data pretreatment*’ section on page 11 of the *Revised Manuscript with Track Changes*.

2. This suggests that not all pertinent facets of rural financial risk may have been sufficiently covered by the statistical techniques employed in the investigation. It implies that a more comprehensive or in-depth approach to the investigation would be advantageous.

**Response:** Thanks for your valuable suggestions. However, we aim to construct the rural financial index using the entropy weight method at the macro level and analyze the regional characteristics of rural financial risk in China at the meso level by employing tests such as Moran index, Dagum Gini coefficient, and three-dimensional kernel density estimation. The next step of our research will involve utilizing more comprehensive and in-depth investigation methods like spatial econometric models to examine the spatial spillover effects of China's rural financial risks and identify their underlying causes, which is also the limitation of this paper. Please refer to the adding part *Limitations* on page 26 of the *Revised Manuscript with Track Changes*.

3. The recommendation to use more complex statistical tools, such spatial econometrics or time-series analysis, suggests that these approaches may provide a more comprehensive and nuanced understanding of financial risk in rural areas. While spatial econometrics takes into account the geographical interactions between variables in addition to their temporal dynamics, time-series analysis concentrates on analysing data points gathered over time to detect trends and patterns.

**Response:** Thanks for your useful comments and valuable suggestions! However, the primary objective of this paper is to construct the rural financial risk index using the entropy weight method, in order to conduct a series of empirical studies on the spatial perspective of the risk index. This paper does not address the relationships among explanatory variables, explained variables, and control variables as they are beyond the scope of this research. Analyzing the factors influencing rural financial risk levels from a spatial measurement perspective will be addressed in our future research endeavors.

4. Using more advanced statistical methods aims to produce a more reliable evaluation of financial risk in rural areas. Robust assessments are more likely to be accurate, thorough, and dependable, which might result in better policy recommendations and decision-making.

**Response:** Thank you for your very valuable comments again! Following the research of Liu et al. [1], we focus on the stable development of rural finance in China and categorizes the research subject into rural financial risk index. After conducting a systematic analysis and testing for changes and spatial differences in risk levels across various regions in China, suggestions are provided for promoting coordinated development in rural areas. Therefore, the research content of this paper does not involve the use of spatial econometric model to construct explanatory analysis among variables, let alone the robust assessments among variables, which will be addressed in our future work, but beyond the scope of this paper.

5. The study's possible data constraints are not sufficiently addressed in the report. It is crucial to recognize and address any data constraints that can have an impact on the validity and generalizability of the findings given the complexity of rural financial risk.

**Response:** Thank you for your valuable comments! First of all, we refer to relevant research references for the selection of data indicators, as well as relevant documents released in combination with China's national conditions, the methods employed to select data and construct China's rural financial risk index are deemed appropriate. Of course, in view of the complexity of rural financial risk, the data of this paper is limited to a certain extent, please refer to the description about *Limitations* part on page 26 of the updated *Manuscript*. Nevertheless, it enhances the analysis of theory by providing a comprehensive description of the selected indicators, please see the part of *Indicators selection and data pretreatment* on page 11 of the corrected manuscript. Additionally, we have been supplemented and uploaded relevant data information which can be found the supplemented indicators (*x*_4_, *x*_9_, *x*_13_) description of Table 2 on pages 14-15 and data *Supporting Information* on page 27 of the revised *Manuscript*.

6. The literature review is shallow and superficial. To demonstrate the importance of their study in the larger academic framework, the authors ought to present a more thorough analysis of the body of work already written on rural financial risk in China, taking into account current advancements and discussions in the area.

**Response:** Thank you for your insightful comments. Our answer is YES! We have been tried our best to improve and enhance the analysis of literature review, which is divided into four parts, namely *Study on rural financial risk*, *Financial risk influencing factors*, *Assess rural financial risk*, and *Discussion*. Please refer to the promoted red text in the *Literature Review* section on page 4 of the *Revised Manuscript with Track Changes*.

7. The analysis's results are unduly basic and fall short of capturing the nuances of China's rural financial risk. It is recommended that the authors offer more comprehensive and perceptive analyses of their results, including the wider socioeconomic and policy ramifications.

**Response:** Thank you for your valuable comments and insightful suggestions! We couldn’t agree more! However, we have been re-analyzed the empirical part of the *Empirical research* section, and tried our best to enhance the analysis of the results, thereby providing more comprehensive and insightful results.

8. It might be challenging for readers to understand the points made because of the writing style, which is frequently ambiguous and dense. In order to ensure that the main arguments are expressed clearly and succinctly, the authors should rewrite the document.

**Response:** Thank you very much! We have been read the whole paper again, and tried our best to make the paper stronger, including the language style and check whether some sentences are used properly. Please refer to the red text in the *Revised Manuscript* for the modified and supplementary content.

9. The study's theoretical foundation is not well defined or stated. The conceptual framework and theoretical presumptions that underpin the authors' analysis should be spelt out in detail, providing a more solid theoretical basis for the research.

**Response:** Thank you for your useful comments again! Following the researches of Liu et al. [1], Cheng and Yang [2], our paper’s research paradigm is strictly in accordance with the writing norms of the risk index in the study area, which doesn’t involve the proposal of hypotheses. It should be noted that this paper aims to comprehensively test the rural financial risk index rather than testing specific hypotheses. Furthermore, considering that some theoretical basis and some definitions are difficult for readers to read at the beginning, we provide additional supplementation of theoretical basis primarily within its *Introduction*, *Literature Review*, and *Empirical Analysis*. Please refer to the red text of the *Revised Manuscript with Track Changes*.

**References**

[1] Liu FG, Liao ZJ, Zhang M. Research on the Spatial Distribution and Evolution Characteristics of Regional Financial Risks in China. *Journal of Yunnan University of Finance and Economics*. 2022; 38(4): 66-84. doi:10.16537/j.cnki.jynufe.000780. (in Chinese)
[2] Cheng HX, Yang L. The Study of Spatial Distribution and Diffusion of New Rural Financial Institutions: Based on ArcGIS Software. *Economic Geography*. 2020; 40(2): 163-170. doi:10.15957/j.cnki.jjdl.2020.02.018. (in Chinese)

**Responses to the Reviewer #3**

The paper presents an interesting analysis of China’s rural financial risk, focusing on its regional differences, spatial distribution, and dynamic evolution, based on data from 2009 to 2021, covering 30 Chinese provinces. The literature review is relevant and well conducted, the research results are clearly presented, and the topic is interesting for the journal’s audience. However, the credibility of the findings is affected by the fact that the data underlying the conclusions have not been provided by the authors. In my opinion, there are some issues that need to be addressed and improved.

**Response:** Thank you for your highly appreciation and valuable comments. After a careful major revision, we have made this paper much stronger.

**Specific comments:**

1. Please explain all the symbols used in the equations of section 3. In the present form, the equations are difficult to follow and understand on first reading.

**Response:** Yes, thank you for your valuable suggestions! We have been enhanced and supplemented the explanation of formulas in the Methods section, please see the red text of the Methods part on pages 7-11 of the *Revised Manuscript with Track Changes*.

2. I noticed that indicators x4, x9 and x13 are missing from the analysis in Table 2 and if I understood correctly from the explanations following Table 2, these indicators did not exhibit varying weight proportions within the rural financial risk index across the 30 analyzed provinces. However, it is not clear whether these indicators were taken into account in the calculation of the rural financial risk index? Were they relevant for this calculation? Please explain.

**Response:** Thank you very much! We have been added the values of x4, x9 and x13 to Table 2, these indicators are indeed taken into account when calculating the rural financial risk index, they were not shown in the Table 2 before because of paper formatting issues. At present, we have been supplemented the explanation of Table 2, please refer to the red text on pages 14-15 of the *Revised Manuscript with Track Changes* for details.

3. What are the limitations of the study? Provide a brief description of these limitations before the conclusions section.

**Response:** Yes, thanks for your valuable suggestion! We have been supplemented the research limitations of this paper in the section “*Limitations*.” Please see the red text descripted in the section “*Limitations*” on page 26 of the *Revised Manuscript with Track Changes* for details.

4. According to the journal’s publication criteria, authors must follow standards and practices for the availability of data. According to your submission letter, “all data is fully available without restriction” in the manuscript and its Supporting Information files. However, I was unable to find any available data that could support the results and conclusions of the paper based on the indications of the manuscript. Please refer to the PLOS Data Policy and address this issue, as it is very important to comply with the journal’s publication criteria.

**Response:** Thanks again! In accordance with the journal’s publication criteria, we have been uploaded relevant datasets supporting the conclusions of this paper, please refer to *S1 Data.* and *S2 Data.* of the Supporting information on page 27 in the *Revised Manuscript with Track Changes*.
